# Potato Peels Mediated Synthesis of Cu(II)-nanoparticles from Tyrosinase Reacted with bis-(*N*-aminoethylethanolamine) (Tyr-Cu(II)-AEEA NPs) and Their Cytotoxicity against Michigan Cancer Foundation-7 Breast Cancer Cell Line

**DOI:** 10.3390/molecules26216665

**Published:** 2021-11-03

**Authors:** Akbar Idhayadhulla, Aseer Manilal, Anis Ahamed, Saud Alarifi, Gurusamy Raman

**Affiliations:** 1 Research Department of Chemistry, Nehru Memorial College, Affiliated Bharathidasan University, Puthanamapatti 621007, Tamil Nadu, India; 2 Department of Medical Laboratory Science, College of Medicine and Health Sciences, Arba Minch University, Arba Minch P.O. Box No. 21, Ethiopia; aseermanilal@gmail.com; 3 Department of Botany & Microbiology, College of Sciences, King Saud University (KSU), P.O. Box 2455, Riyadh 11451, Saudi Arabia; nanisahamed@gmail.com; 4 Department of Zoology, College of Sciences, King Saud University (KSU), P.O. Box 2455, Riyadh 11451, Saudi Arabia; salarifi@ksu.edu.sa; 5 Department of Life Science, Yeungnam University, Gyeongsan 38541, Gyeongbuk-do, Korea; bioramg@gmail.com

**Keywords:** copper nanoparticles, MCF-7 breast cancer cell, cytotoxicity screening, DNA fragmentation, fluorescence microscopy

## Abstract

The synthesis of nanoparticles is most important in the context of cancer therapy, particularly copper nanoparticles, which are widely used. In this work, copper(II)-tyrosinase was isolated from potato peel powder. Copper nanoparticles (Tyr-Cu(II)-AEEA NPs) were synthesized via the reaction of tyrosinase with *N*-aminoethylethanolamine to produce Cu(II)-NPs and these were characterized by means of FT-IR, UV-Spectroscopy, XRD, SEM, TEM and a particle size analyzer. These Tyr-Cu(II)-AEEA NPs were tested as anticancer agents against MCF-7 breast cancer cells. Fluorescence microscopy and DNA fragmentation were also performed, which revealed the inhibiting potentials of Cu(II)-AEEA NPs and consequent cell death; Tyr-Cu(II)-AEEA NPs show potential cytotoxicity activity and this nano material could be contemplated as an anticancer medicament in future investigations.

## 1. Introduction

In recent years, nanotechnology has become a promising research field and its current focus on medicine and nanoparticles (NPs) has an immense scientific attraction in the context of biological screening. Currently, the cases of cancer are enhanced due to various factors and the World Health Organization (WHO) reported that the incidence of cancer increased from the year 2008 and now it reaches above 12.7 million, which is equivalent to 25 million per year [1]. In this emergency situation, several researchers are working in this area, but the exact causes have not been identified and there exists the need for novel anticancer drugs with zero side effects. Recent developments in nanotechnology-based anticancer drugs [2,3], for example, Abraxane, Doxil, Myocet, correspond to a medical breakthrough, along with chemotherapy, surgery, and radiotherapy [4]. The most common type of cancer in females is breast cancer, as per the latest records of global cancer statistics. A major procedure for eliminating cancerous cells is through radiotherapy by applying ionizing radiations [5]. High radiation doses reduce the growth of malignant cells [6]. Several studies have attempted to solve this conundrum through the development of a breast cancer therapy via chemical or thermal agents [7]. NPs have a wide range of applications; nevertheless, they are known to pose serious negative impacts on living organisms, starting from the site of its production [8]. For instance, they can cause inflammations and fibrosis in multicellular organisms and cytotoxicity in unicellular beings. Particularly in mammals, NPs are absorbed by endocytosis and stored in different sites including mitochondria, subsequently causing toxic effects including oxidative stress. In humans, they can inflict a series of respiratory and cardiovascular diseases. Some of them are even categorized as carcinogens (e.g., polycyclic aromatic hydrocarbons) [9]. It has also been reported that air polluted with NPs can cause the death of laborers in gas, coal and asphalt industries [10]. Cancer nanotechnology is a highly promising frontier area of research including multiple branches of science, engineering and medicines with wider applications and varieties of NPs. The basic rationale behind this type of work is that new NPs are expected to be useful in molecular diagnosis and targeted therapy in connection with cancer treatment. Among them, copper is one of the most required elements, particularly because of the stability it provides to the tissues and in the formation of collagen. Tyrosinase has a di-nuclear copper active center, and it catalyzes the hydroxylation and subsequent oxidation reactions that convert phenol to related orthoquinone as well as the oxidation of catechol to quinone [11,12]. A large number of reports show that copper oxide nanoparticles are used for the screening of MCF-7 breast cancer cells [13,14]. In some of the previous reports, it is mentioned that CuO NPs were effective in the cytotoxicity screening and Deoxyribonucleic acid A damage tests (A549 and K562) [15]. We report here the synthesis of copper-coated bis-(*N*-aminoethylethanolamine) NPs, and the morphology of bis-(*N*-aminoethylethanolamine)- Cu-NPs evaluated via various techniques. Notably, the anticancer effects of varied concentrations of Cu-NPs were appraisable on Michigan Cancer Foundation-7 (MCF-7) breast cancer cells, as per the MTT ((3-(4,5-dimethylthiazol -2-yl)-2,5-diphenyltetrazoliumbromide)tetrazolium reduction) assay. This study examines the impacts of Cu-NPs on the viability of active agents against MCF-7 cell lines. The results of an evaluation of anticancer activity using an MTT assay, fluorescence microscopy, and apoptosis assay are also described.

## 2. Results and Discussions

### 2.1. Chemistry

#### 2.1.1. Extraction of Tyrosinase from Potato Peel

**Figure 1:**
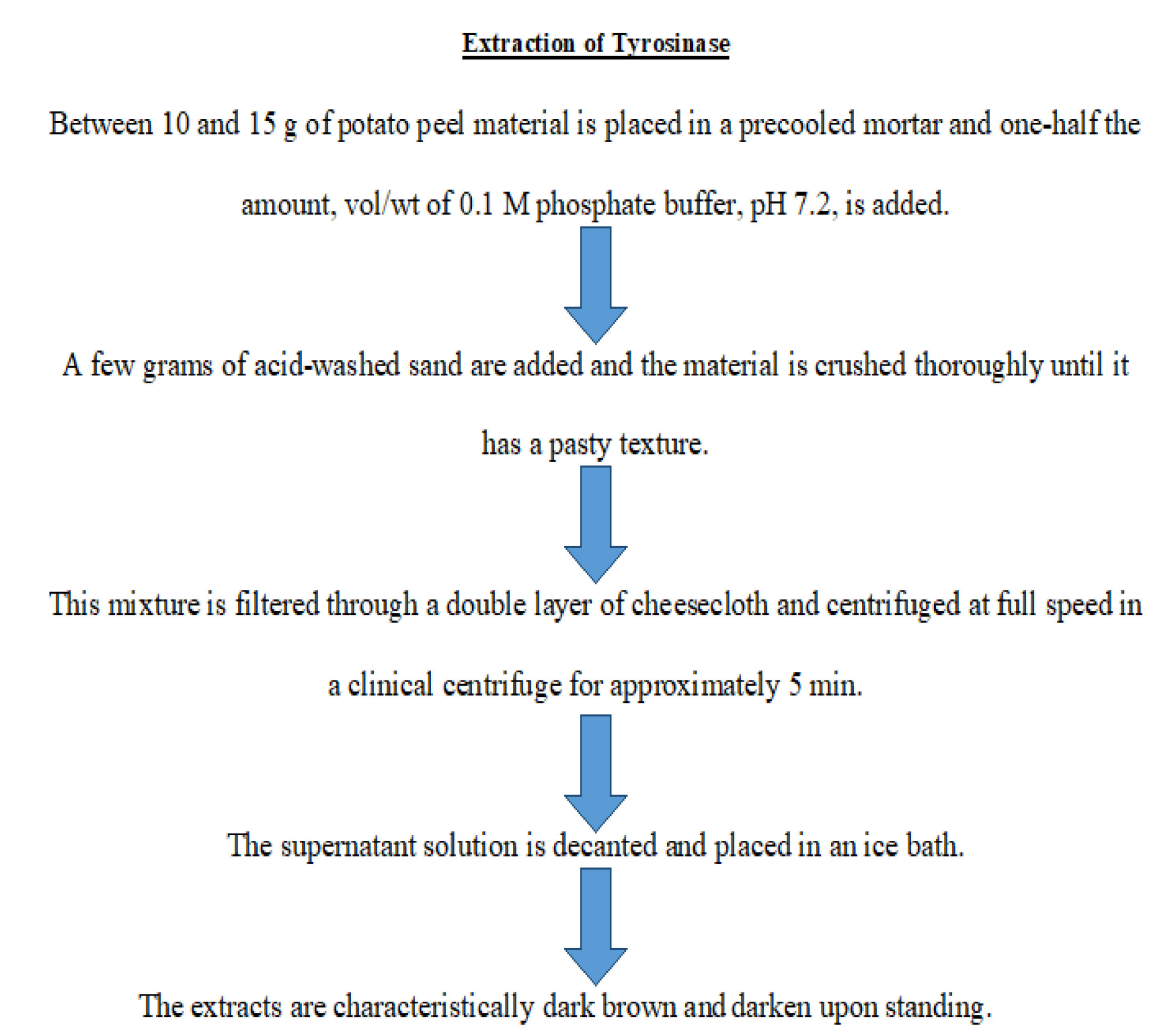
Flowchart for the preparation of potato peel tyrosinase enzyme.

An extract of tyrosinase was obtained from a potato peel and the preparative route is presented in Figure 1.

#### 2.1.2. Preparation of Tyr-Cu(II)-AEEA NPs

**Scheme 1:**
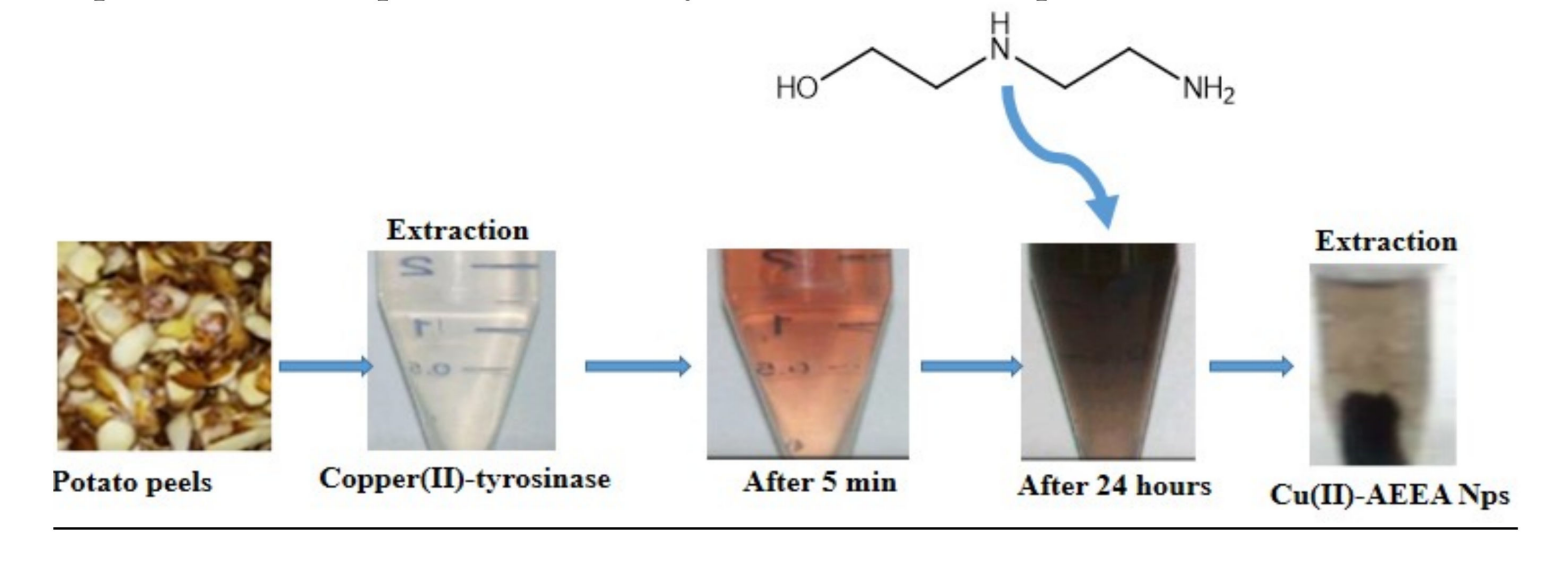

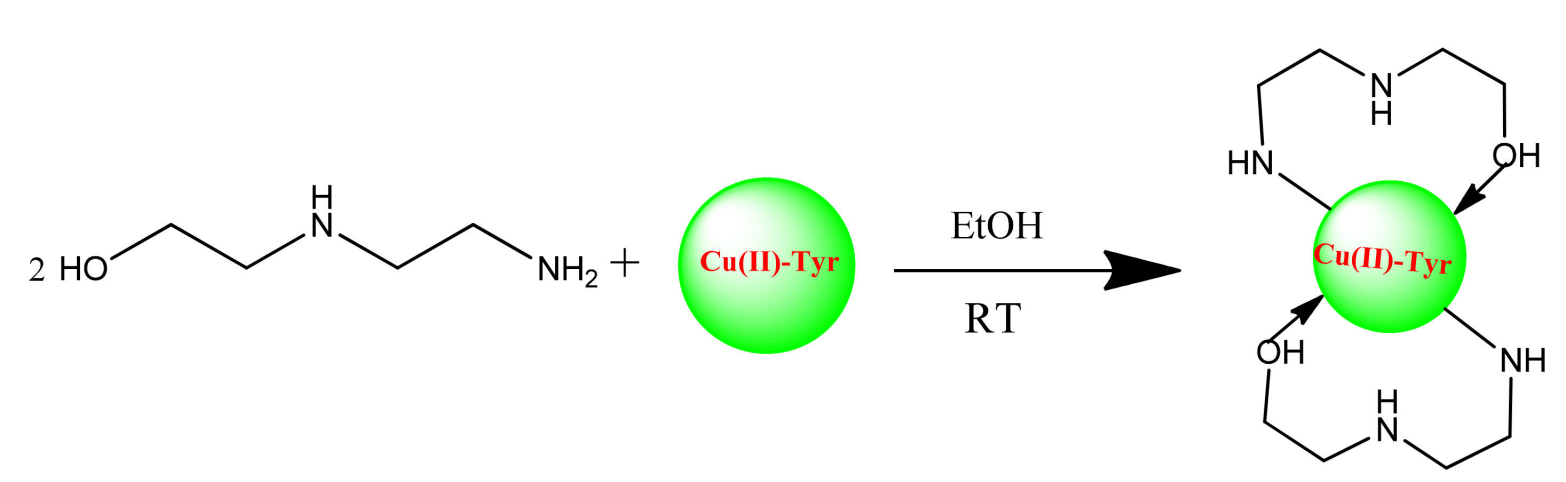
One-pot two component synthesis of bis-(*N*-aminoethylethanolamine)-Copper (II) (Tyr-Cu(II)-AEEA NPs).

The synthesis of bis-(N-aminoethylethanolamine)-Copper(II) nanoparticles (Cu(II)-AEEA NPs) and the reaction outline are represented in Scheme 1. The tyrosinase was mixed with ligand 2-((2-aminoethyl)amino)ethanol at room temperature. The final product formed was a black precipitate and it was filtered for further use and characterization using Fourier transform infrared spectroscopy (FT-IR), Ultraviolet (UV), scanning electron microscopy (SEM), tunneling electron microscopy (TEM), X-Ray diffraction technique (XRD), and particle size analysis to confirm the particle size.

#### 2.1.3. UV–Visible Structural Analysis

**Figure 2:**
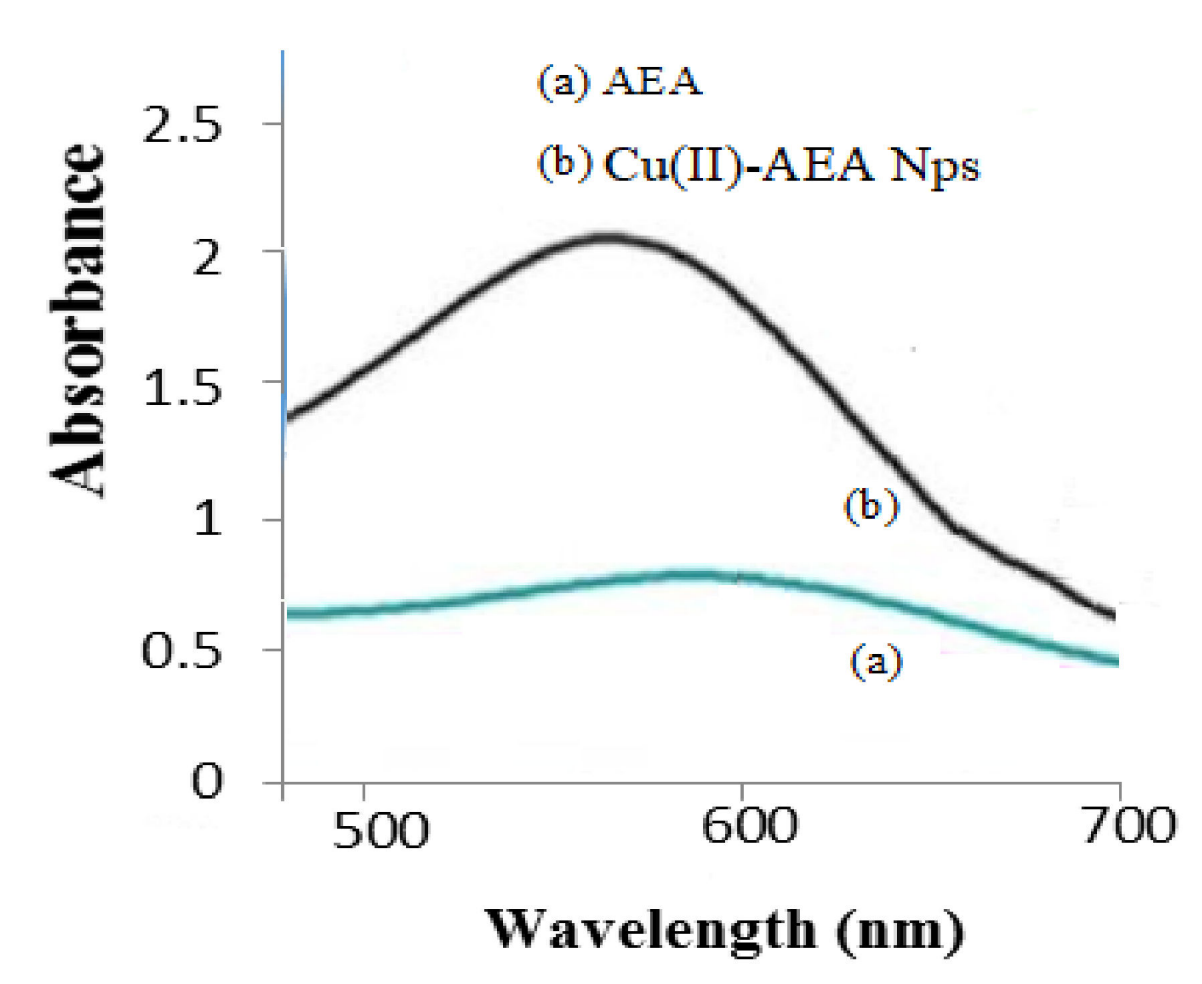
UV–vis spectra of Cu(II)-AEA NPs.

Cu nanoparticles were formed from the interaction of AEEA ligand with Cu(II) Tyrosinase. The Tyr-Cu(II)-AEEA NPs absorption was recorded at 556 nm, whereas the AEEA ligand showed no peak between 500–700 nm. The UV−visible spectra are shown in Figure 2. 

#### 2.1.4. FT-IR Spectra 

**Figure 3:**
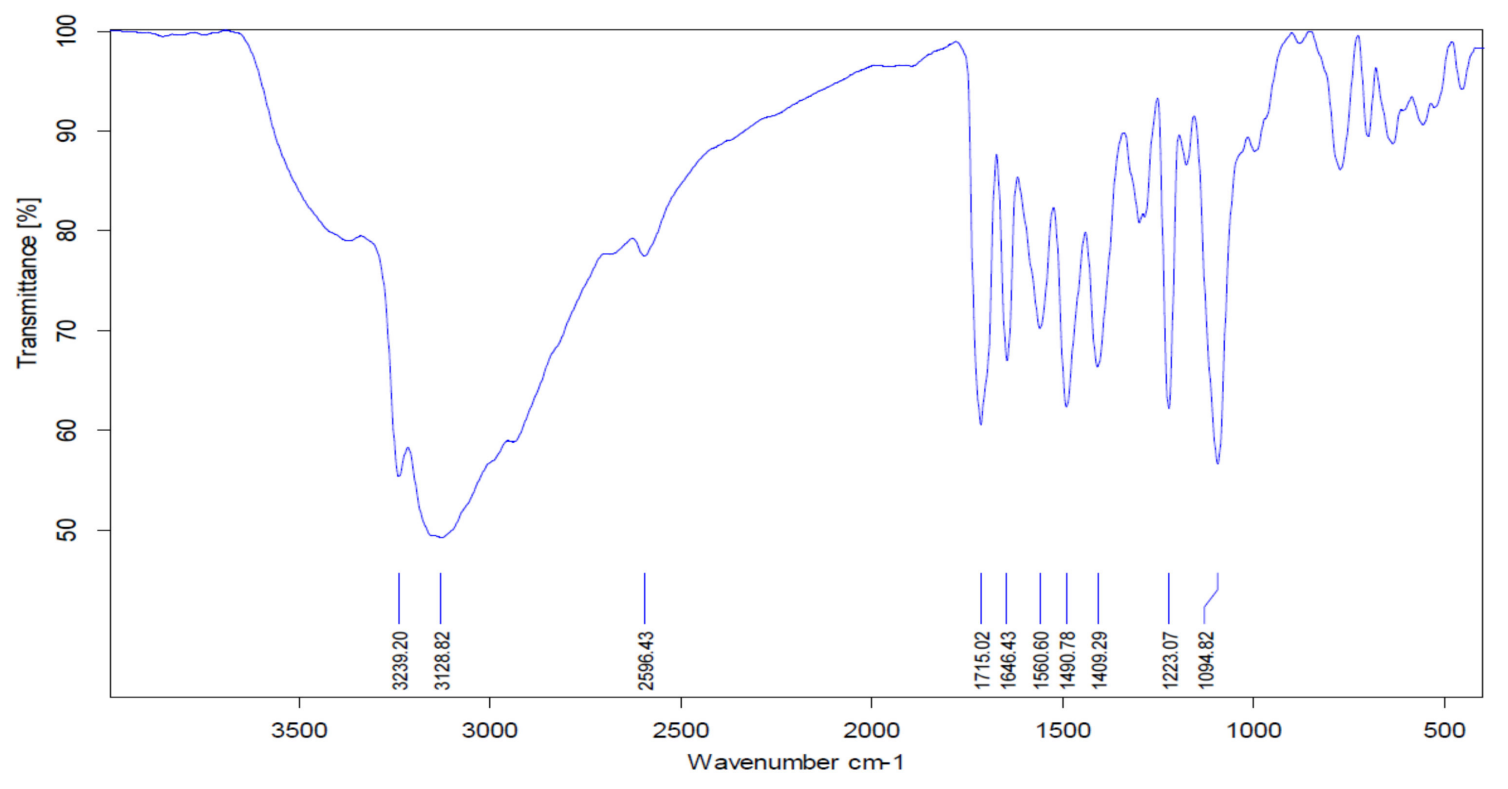
IR spectrum of Tyr-Cu(II)-AEEA NPs.

The main characteristic vibrations of Tyr-Cu(II)-AEEA NPs obtained from FT-IR analysis are shown in Figure 3. The IR vibrational values from the literature were referred to analyze the vibrational frequency of the functional group present in the compound. The O−H stretching in the center of Cu, was assigned to 3239 cm^−1^ for Tyr-Cu(II)-AEEA NPs. The 1223.07 cm^−1^ band corresponds to the C-H stretch of aliphatic amines not connected to Cu. The absorption bands at 1094.82 and 1409.29 cm^−1^ are due to the N-H bend in the primary amine (the unreacted ligand). The absorption band at 843 cm^−1^ corresponds to N-H out of plane bending, mostly due to aliphatic secondary amines; here, this secondary amine was formed by the reaction between the ligand and Cu-(II)-Tyr, the N-H bend of primary amine (scissoring) corresponds to the peak at 1560.6 cm^−1^. This shows that some unreacted ligands exist, along with the product formed.

#### 2.1.5. Particle Size Analysis 

**Figure 4:**
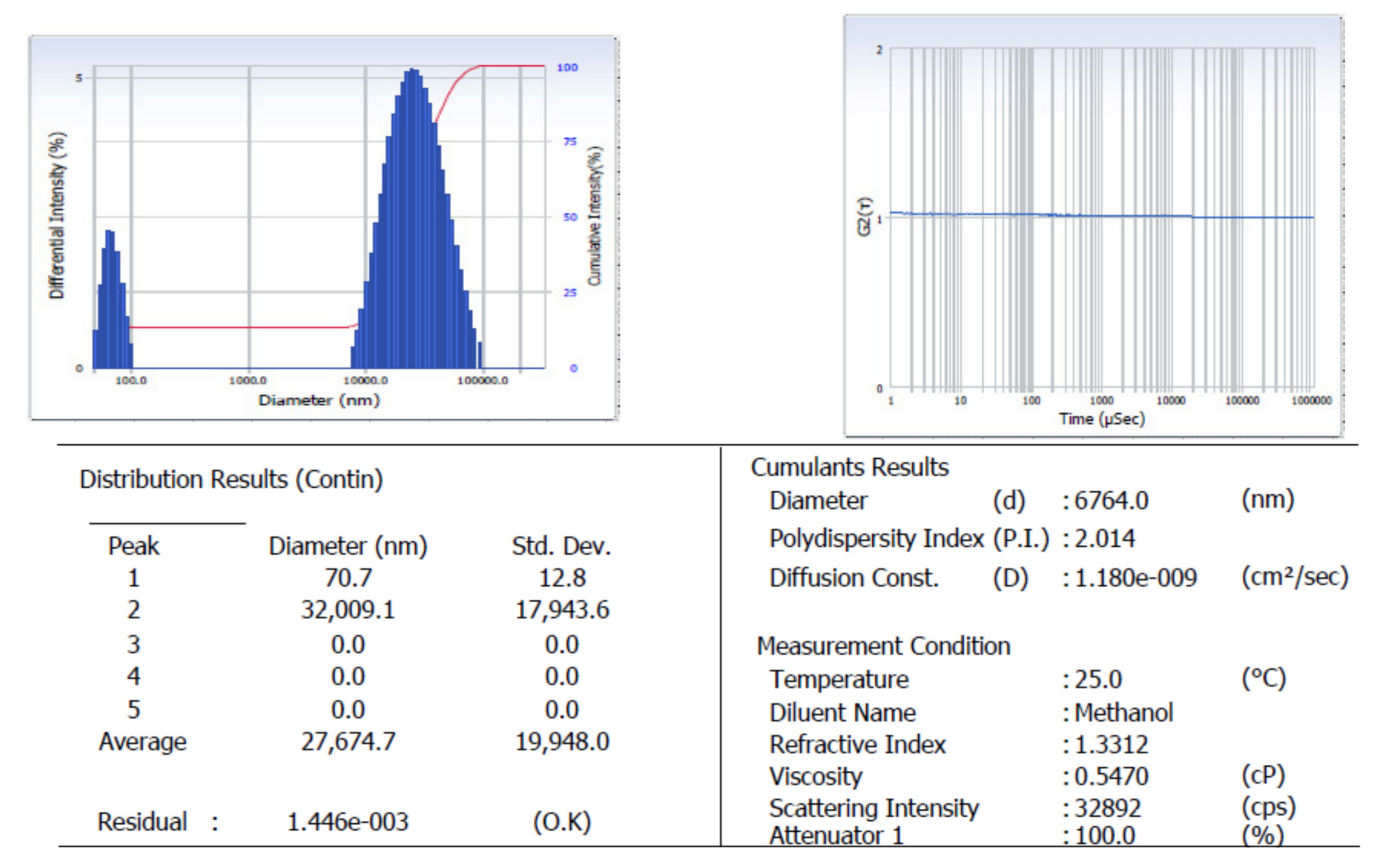
Particle size distribution of Cu(II)-AEA NPs.

The particle size analyzer was measured by scrutinizing the particle size distribution curve and surface charge of the Tyr-Cu(II)-AEEA NPs. Figure 4 indicates that the first peak represents a 70.7-nanometer diameter and a standard deviation of 12.8, which corresponds to 100 numbers of average size nanoparticles present in the target nano material. Figure 4 shows the intensity vs. the diameter of the first peak of Cu-NPs, and it also reveals the existence of Tyr-Cu(II)-AEEA NPs, which indicate a particle size of 110 nm with a narrow index. Therefore, Tyr-Cu(II)-AEEA NPs are suitable for therapeutic applications in cancer treatment due to the exact particle size (<100 nm).

#### 2.1.6. SEM Analysis 

**Figure 5:**
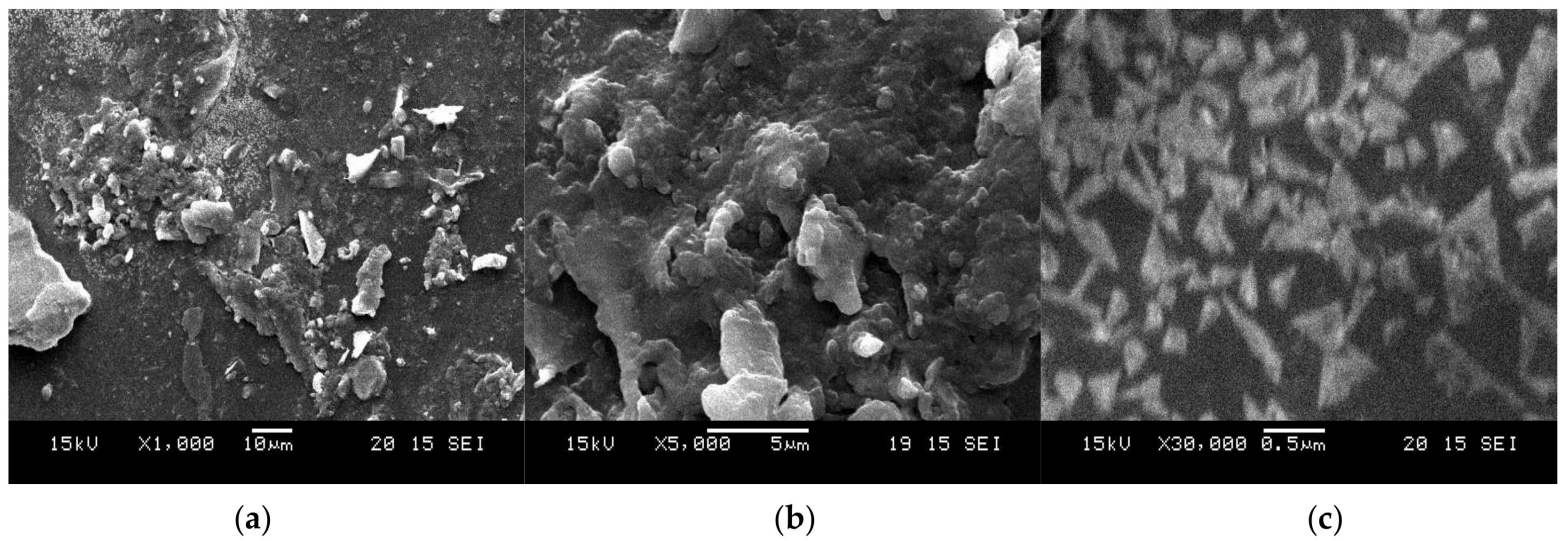
Tyr-Cu(II)-AEEA NPs of SEM images. SEM-EDAX images of Tyr-Cu(II)-AEEA NPs obtained (**a**) at 10 µm, (**b**) at 5 µm, and (**c**) at 0.5 µm in a water mixture.

Figure 5 illustrates the SEM-EDAX images of Tyr-Cu(II)-AEEA NPs obtained (a) at 10 µm, (b) at 5 µm, and (c) at 0.5 µm in a water mixture. These images also confirm the formation of nanoparticles and the presence of elements such as Cu, C, O, and N.

#### 2.1.7. Powder X-ray Diffraction Studies 

**Figure 6:**
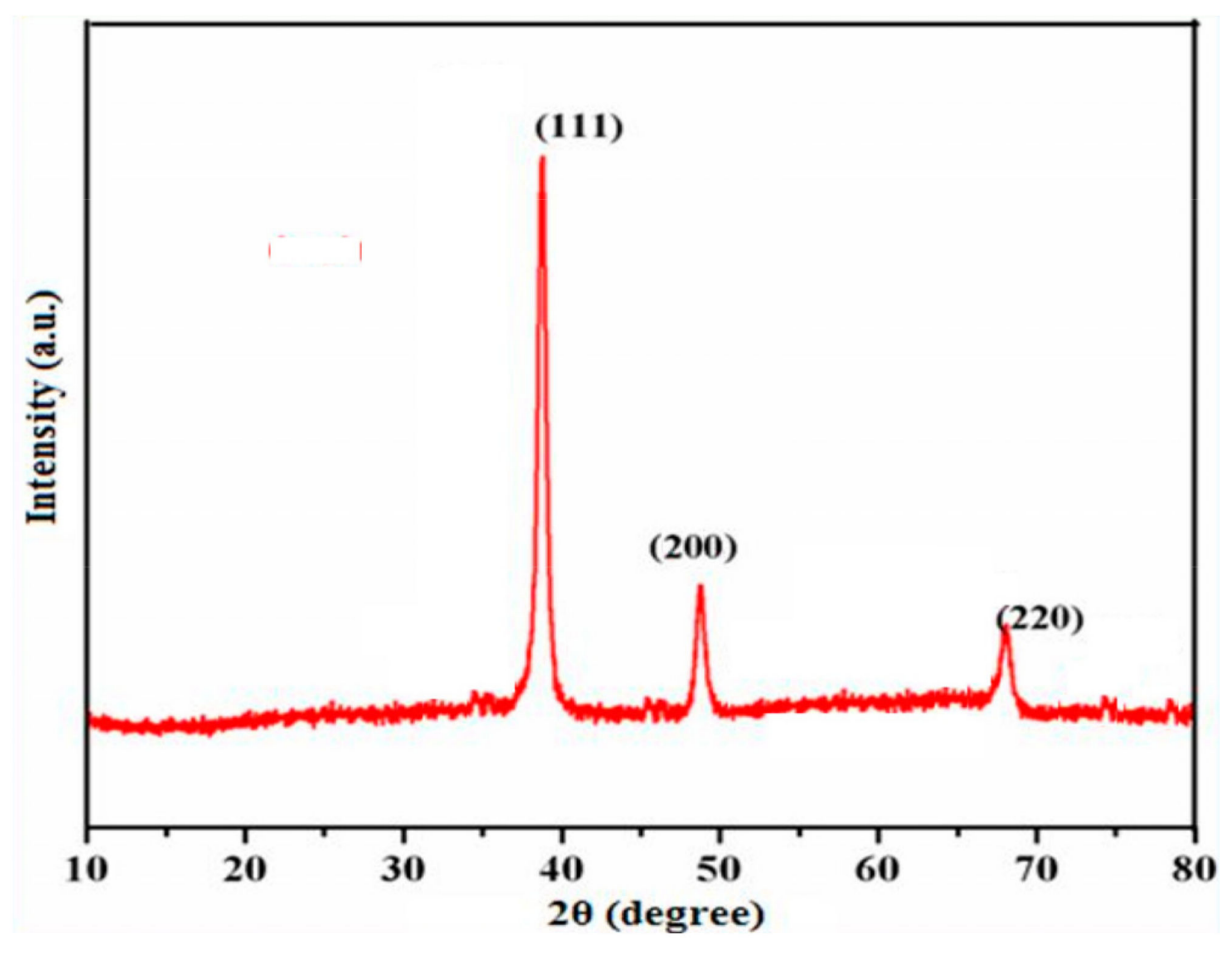
XRD Studies of Cu(II)-AEA NPs mediated copper nanoparticles.

The structural analysis and phase crystallinity of the synthesized Tyr-Cu(II)-AEEA NPs were examined by means of a powder X-ray diffraction method. Figure 6 signifies the diffraction pattern of Cu nanoparticles at 2θ (two theta) value of 45.10°, 49.92° and 73.10° with respect to (111), (200) and (220) planes independently. Additionally, it confirmed the copper cubic lattice formation; JCPDS No. 040836 indicates a good agreement in line with the centered cubic phase.

#### 2.1.8. TEM Image Analysis 

**Figure 7:**
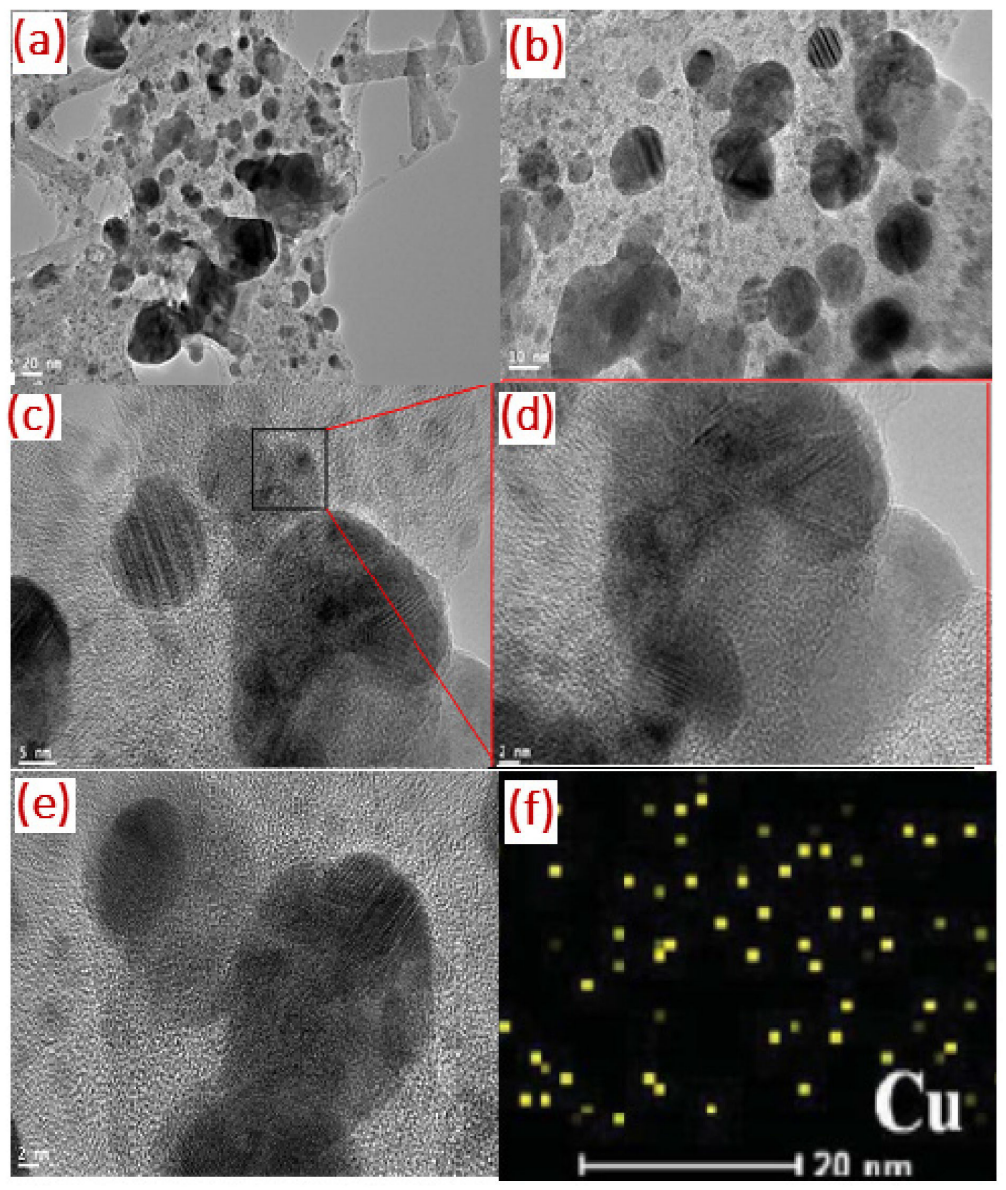
TEM image of Tyr-Cu(II)-AEEA NPs at (**a**) 20 nm, (**b**) 10 nm, (**c**) 5 nm, (**d**) HRTEM image, (**e**) lattice fringes of Cu(II)NPs, along with elemental maps of (**f**) Cu kα shell.

The size and morphology of the as synthesized nanoparticles were observed using TEM. Figure 7a–c present TEM images of Tyr-Cu(II)-AEEA NPs at 20 nm, 10 nm, and 5 nm, respectively; in the High Resolution TEM image, the bright/dark contrast corresponds to the Cu nanostructure, and is attracted to the exterior in the presence of oxygen atoms.

### 2.2. Anticancer Activity 

The nanoparticles Tyr-Cu(II)-AEEA NPs were assessed for anticancer activity against MCF-7 (Human breast cancer), by using an MTT assay [16]. The percentages of inhibition are presented in Table 1. The results revealed that these nanoparticles displayed potential anticancer activity with IC_50_ (inhibitory concentration) values ranging from 9 to 76% against the MCF-7 cancer cell line, in destroying the breast cancer cell line, an MCF-7 with IC_50_ value of 55.31 µg/mL. Hence, the MCF-7 cell line was selected as a model for the subsequent experiments.

#### 2.2.1. Apoptosis Assay

**Figure 8:**
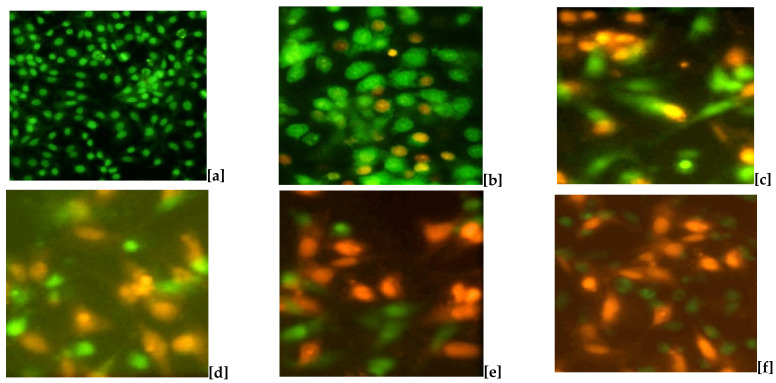
Apoptotic effect of Tyr-Cu(II)-AEEA NPs on MCF-7 cells. (**a**) Cell control, (**b**) 5 µg/mL, (**c**) 25 μg/mL, (**d**) 50 μg/mL, (**e**) 75 μg/mL, (**f**) 100 μg/mL.

A cytotoxic assay of Tyr-Cu(II)-AEEA NPs was carried out using the double staining method on MCF-7 cell lines. The number of identifications shown in Figure 8 correspond to cell performances such as nil death of cells for green fluorescence and loss of cell membrane permeability for orange fluorescence. The treatment of control (a) shows no cell death; it is indicated by the full green image; however, a single nano particle was used for the screening at a concentration of 5 µm, which shows that some of the orange color appeared, marking the initiation of cell death. According to the increase in the concentration of the test sample Tyr-Cu(II)-AEEA NPs, the death percentage went up to 100 µm and no green fluorescence was observed, hinting that all the cell lines were dead at this condition. Therefore, the nano material of Tyr-Cu(II)-AEEA NPs can be considered very effective. 

#### 2.2.2. DNA Fragmentation Analysis

**Figure 9:**
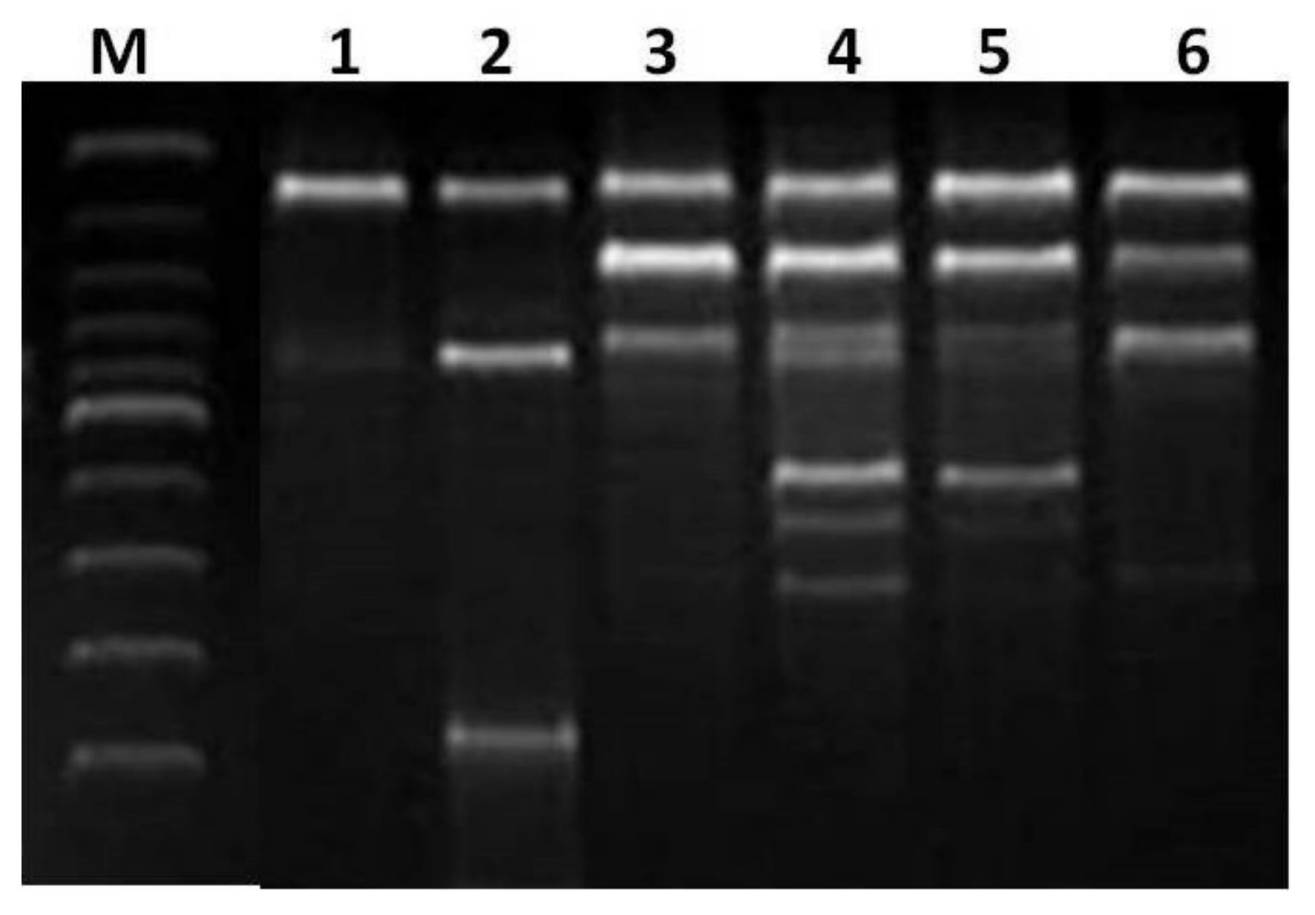
DNA fragmentation of Tyr-Cu(II)-AEEA NPs with a 5–100 μm/mL concentration in MCF-7 cell line M: 100 Bp DNA ladder; 1: Untreated DNA; 2: 5 μg/mL treated; 3: 25 μg/mL treated;. 4: 50 μg/mL treated; 5: 75 μg/mL treated; 6: 100 μg/mL treated.

Cu(II)-AEEA NPs were studied by DNA fragmentation via oligonucleosomal DNA fragmentation (DNA ladder), and the method corresponds to a previously reported work [17]. The programmed cell death or apoptosis was followed, as cited in a previous work [18]. MCF-7 cells were treated with Tyr-Cu(II)-AEEA NPs at a 5–100 μg/mL concentration for 48 h. The DNA was run on 2% agarose gel electrophoresis after staining with ethidium bromide and observed under UV illumination. The Tyr-Cu(II)-AEEA NPs were detected with DNA fragmentation, as shown in Figure 9. The DNA fragmentation technique was used to understand the endonuclease cleavage products of apoptosis. It was revealed that the DNA fragmentation pattern in the cells treated with NPs can be correlated to death. 

## 3. Materials and Methods

All the chemicals and reagents were purchased from Merck (Whitehouse Station, NJ, USA). The FT-IR spectra (4000–400 cm^−1^) were recorded in KBr on a Shimadzu 8201pc (Shimadzu Ltd., Kyoto, Japan). 

### 3.1. Preparation of Cu(II)-Tyrosinase Enzyme 

The enzyme was prepared as per the methodology previously reported [19]. Briefly, potato peels (~10 g) were ground to a paste with 50% (*v/w*) 0.1 M phosphate buffer (pH 7.2) and a few grams of acid-washed sand, in a cooled mortar. The paste was filtered over a double layer of cheesecloth, and the filtrate was separated using centrifugation at full speed in a clinical centrifuge for 5 min. The supernatant solution was decanted and placed in an ice bath.

### 3.2. Synthesis of Tyr-Cu(II)-AEEA Nanoparticles

The prepared mixture of tyrosinase and *N*-aminoethylethanolamine (1 mmol) in ethanol were added to a mortar (Grindstone stone) at room temperature and pounded for up to 15 min. The obtained solid was mixed with 1 M NaOH. After the completion of the reaction, the synthesized nanoparticles were dried and washed with a suitable solvent and the final product obtained was blue in color.

### 3.3. Cytotoxicity Screening 

#### 3.3.1. Drug Preparation 

The sample Tyr-Cu(II)-AEEA NPs preparation was performed as per our previous study [20].

#### 3.3.2. Cell Viability Test

The cell viability test was conducted by using an MTT (3-(4,5-dimethylthiazol-2-yl)-2,5-diphenyltetrazolium bromide) assay in MCF-7 cell lines, according to the method described in a couple of publications [20,21].

#### 3.3.3. Cytotoxic Assay (MTT Method)

Tyr-Cu(II)-AEEA NPs were subjected to cytotoxic screening as per the methodology described in our previous study [20]. To mention the procedure briefly, viable cells were counted and seeded distinctly in 96-well plates at a density of 1×10^5^ cells per well. After 24 h, cells were washed twice with 100 μL of serum-free medium and starved for an hour at 37 °C. After starvation, cells were treated with different concentrations of test samples (1–100 µg/mL) for 24 h. Blank samples (medium only) served as the control. At the end of the treatment period, the medium was aspirated and serum-free medium containing MTT (0.5 mg mL^–1^) was added and incubated for another 4 h at 37 °C in a CO_2_ incubator. The MTT containing medium was then discarded and the cells were washed with PBS (200 μL). The formed formazan crystals were then dissolved by adding 100 μL of DMSO. Spectrophotometric absorbance of purple blue formazan dye was measured in a microplate reader Bio-Rad 680 (Bio-Rad Laboratories, Inc. Hercules, CA, USA) at 570 nm. Cytotoxicity (IC_50_ values) was determined using GraphPad Prism software.

#### 3.3.4. Fluorescence Microscopic Analysis of Apoptotic Cell Death

In this study, an acridine orange/ethidium bromide (AO/EB) double staining assay was used as recommended by a previous study [22]. Acridine orange is taken in viable and nonviable cells as well as ethidium bromide is taken in nonviable cells; both emit red fluorescence by intercalation into DNA. This procedure corresponds to the method described in the literature [23]. Sample preparation: 20 μL of dye mixture (AO and EB) in distilled water was mixed with the cell suspension in a 96-well plate and after the incubation time, nano materials Tyr-Cu(II)-AEEA NPs were added immediately and viewed through the Olympus inverted fluorescence microscope (Nikon Eclipse TS200-U, Tokyo, Japan) at 200× and 400× magnification. 

#### 3.3.5. DNA Fragmentation

In a 24-flat-wells plate, Tyr-Cu(II)-AEEA NPs were incubated 2 × 10^5^ cells with different concentrations of test material. Fresh DMEM medium was added and allowed to incubate for 24 h; the cell samples were collected in a 1.5-milliliter tube, spun down, re-suspended with 0.5 mL of PBS, and 55 µL of lysis buffer was added for 20 min on ice (4 °C). We followed the above protocol according to the method available online (http://hedricklab.ucsd.edu/Protocol/DNAFRAG.html, accessed on 13 November 2019). The image was observed by DNA shearing in a 312-nanometer UV illuminator.

## 4. Conclusions

Copper(II)tyrosinase-bis-(*N*-aminoethylethanolamine) nanoparticles (Tyr-Cu(II)-AEEA NPs) were synthesized and characterized through FT-IR, UV, SEM, X-ray diffraction, TEM, and particle size analysis. Tyr-Cu(II)-AEEA NPs were screened for cytotoxic activity against MCF-7 breast cancer cells. Tyr-Cu(II)-AEEA NPs showed an excellent response and potential activity against the MCF-7 breast cancer cell line; cell death was confirmed via fluorescence microscopic and DNA fragmentation analysis. The underlying principle involved is that new NPs have a definite potential to be used as promising candidates. Obviously, the above-mentioned nanoparticles are very important in the context of further studies for the development of anticancer agents, with minimal side effects.

## Figures and Tables

**Table 1:** Percentage of viability of Cu(II)-AEA NPs.

Test Sample Concentration (µg/mL)	% of Activity
Control	9
1	17
5	22
10	27
25	35
50	46
75	60
100	76

## Data Availability

Data used to support the findings of this study are available from the corresponding author.

## Sample Availability

Samples of the compounds are available from the authors.
